# *Hamamelis virginiana* L. in Skin Care: A Review of Its Pharmacological Properties and Cosmetological Applications

**DOI:** 10.3390/molecules30132744

**Published:** 2025-06-26

**Authors:** Magdalena Wójciak, Wiktoria Pacuła, Ireneusz Sowa, Marcin Feldo, Filip Graczyk, Daniel Załuski

**Affiliations:** 1 Department of Analytical Chemistry, Medical University of Lublin, Chodźki 4a, 20-093 Lublin, Poland; wiktoria.pacula@umlub.pl (W.P.); i.sowa@umlub.pl (I.S.); 2 Chair and Department of Vascular Surgery and Angiology, Medical University of Lublin, 11 Staszica St., 20-081 Lublin, Poland; martinf@interia.pl; 3 Department of Pharmaceutical Botany and Pharmacognosy, Ludwik Rydygier Collegium Medicum, Nicolaus Copernicus University, 9 Marie Curie-Skłodowska Street, 85-094 Bydgoszcz, Poland; filip.graczyk@cm.umk.pl (F.G.); daniel.zaluski@cm.umk.pl (D.Z.)

**Keywords:** witch hazel, hamamelitannin, biological activity, fibroblasts, keratinocytes

## Abstract

*Hamamelis virginiana* L. (witch hazel) is a traditionally used medicinal plant, well-known for its dermatological applications. The plant’s bark and leaves contain a rich array of bioactive compounds, including phenolic acids, flavonoids, catechins, proanthocyanidins, and tannins, many of which exhibit antimicrobial, anti-inflammatory, antioxidant, and wound-healing properties. These activities have been verified by numerous in vitro and in vivo studies, as well as limited clinical trials. The *H. virginiana* extracts have demonstrated effectiveness against bacteria, fungi, and some viruses. Moreover, the extracts exert anti-inflammatory effects by modulating cytokine expression and NF-κB signaling, improve skin regeneration, and protect against UV-induced damage and pollution. This review highlights *H. virginiana* as a complex botanical resource to be used in dermatology and cosmetology and shows that current research offers encouraging results for its future therapeutic use, especially in skin treatment.

## 1. Introduction

*Hamamelis virginiana* L. is a plant from the Hamamelidaceae family, which has been highly valued for years in both medicine and cosmetology. It is native to North America, where it is found along coastal regions, with its primary natural range stretching from southern Canada to the state of Virginia [1]. *H. virginiana* was well known to the native people of North America, who appreciated its medicinal properties [2]. It is commonly known as hamamelis, beadwood, and witch hazel, names which come from indigenous traditions and the plant’s unique seed dispersal mechanism—its ripe fruit capsule opens and shoots seeds at a distance of several meters [3,4].

The leaves and bark of *H. virginiana* have been historically utilized in folk medicine, herbal therapy, and skincare, particularly by Native American communities. Witch hazel extracts are attributed with properties that may help in curing conditions such as psoriasis, eczema, and skin dehydration, as well as easing insect bites, reactions to poison ivy (*Toxicodendron radicans*), and razor burn [2,5]. Additionally, witch hazel has been used for various skin conditions in many European countries, including Poland and Ukraine [6], as well as in the South Balkan and East Mediterranean Region [7,8].

Currently, *H. viriniana* is usually used in medicinal and cosmetic applications. The U.S. Food and Drug Administration (FDA) has approved witch hazel for inclusion in over-the-counter (OTC) formulations. Witch hazel, due to its soothing properties, is a common ingredient in OTC treatments aimed to relieve hemorrhoidal symptoms and vaginal irritations. Furthermore, it has been observed that formulations containing witch hazel extracts have a positive effect on blood vessels and enhance tissue perfusion [9,10]. Witch hazel extracts are widely used in cosmetic products for skincare. They are incorporated into anti-aging creams and gels as well as in cosmetics designed to soothe sunburn-related irritation [11,12,13,14,15]. *H. virginiana*, due to its soothing, anti-inflammatory, and antibacterial properties, is commonly used in products designed to address the irritation of the scalp, including hair tonic and shampoo. It helps reduce sebum production, accelerates the healing of minor wounds, and promotes tissue regeneration [16,17]. For analogous reasons, and due to its non-toxic nature [18], witch hazel is also incorporated into oral hygiene products, including various mouthwashes, toothpastes, and regenerative lip balm [19,20,21]. *H. virginiana* is also applied in wound care management [22]. 

The primary medicinal raw material obtained from *H. virginiana* is its leaves (*Hamamelidis folium*)—dried, whole, or crushed, containing 3–10% of tannin. In addition to the leaves, the bark of *H. virginiana* (8–12% tannins)—referred to as *Hamamelidis cortex*—is also widely used. Both *Hamamelidis folium* and *Hamamelidis cortex* are official herbal drugs listed in the European Pharmacopoeia. Key constituents of the *Hamamelis* plant include ellagitannins, hamamelitannins, flavonoids, gallic and ellagic acids, leucoanthocyanidins, and saponins. These compounds are believed to play a significant role in the cosmetic and medicinal properties of witch hazel preparations. 

Despite its long-standing use and popularity in various therapeutic and cosmetic formulations, robust clinical data supporting its efficacy remain limited. Therefore, the aim of this review is to assess the current state of knowledge regarding *H. virginiana*, with a particular focus on its pharmacological potential in cosmetology. This is to help identify gaps in existing research and highlight the future directions of investigation to validate *H. virginiana*’s medicinal and cosmetic potential. 

A literature survey was conducted using the Scopus, PubMed, ScienceDirect, Web of Science, Springer, and Google Scholar databases. The following keywords were used in the search: “*Hamamelis virginiana*” or “witch hazel”, combined with “skin”, “fibroblast”, “keratinocytes”, “melanocytes”, “antibacterial”, “anti-inflammatory”, “wound healing”, and “anti-aging”. The initial search results were screened by reviewing titles and abstracts for relevance. Full-text articles were then assessed to determine their suitability for inclusion. Studies in which the activity described was not related to dermal application or where the full text was not available in English were excluded from the review. The search period covered the years from 1990 to 2025.

## 2. Botanical and Phytochemical Characterization of *Hamamelis virginiana*

### 2.1. Botanical Characteristic

**Figure 1:**
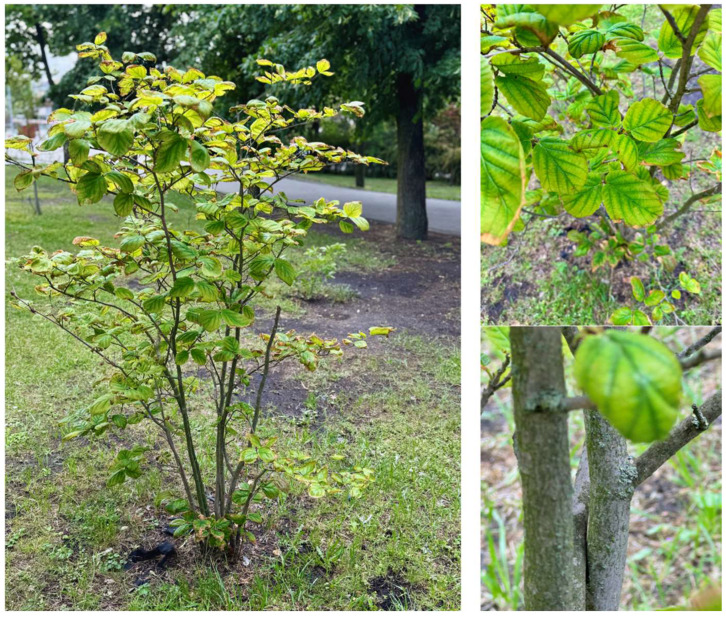
Photo of the *H. virginiana* plant and the parts of the plant used in the treatment of skin disorders. All photos come from the private collection of F. Graczyk and were taken at the Collegium Medicum’s Garden of Medicinal and Cosmetic Plants in Bydgoszcz, Nicolaus Copernicus University (Poland).

*H. virginiana* is a deciduous shrub or tree that typically reaches heights of up to 6 m (Figure 1). 

The plant exhibits significant morphological variability depending on the cultivation area. It often forms dense, multi-stemmed clusters growing from its base. The bark is light brown, smooth, and scaly, with the inner bark displaying a reddish shade. 

The leaves are oval, measuring 3.7–16.7 cm in length and 2.5–13 cm in width, and exhibit an asymmetrical base and a rounded or acute apex, with margins that are wavy-toothed. The petiole is short and stout, measuring 6–15 mm in length. The midrib is more or less hairy, with 6–7 pairs of primary veins. Mature leaves develop a dark green upper surface with a whiter underside. In autumn, the leaves transition to a yellow shade with distinctive rusty spots. Witch hazel blooms between late autumn and winter, occasionally extending to February. The flowers of the witch hazel are pale to bright-yellow, sometimes red or orange, and consist of four ribbon-like petals. They are accompanied by four short stamens and grow in dense collections. Flowering begins in mid-autumn and persists until late autumn. The calyx is four-parted, densely pubescent, and orange-brown on the inner surface. Two or three bractlets are present at the base of each flower. The fruit is a hard, woody capsule, 1.0–1.4 cm long, that matures one year after pollination. When ripening, the capsule rips vigorously at the apex, ejecting two shiny black seeds to distances of up to 10 m from the parent plant [1,23].

### 2.2. Phytochemical Composition 

Despite the recognized medicinal potential of *Hamamelis virginiana* L., relatively few comprehensive phytochemical studies have been conducted, and much of the existing literature remains outdated. Research shows that various parts of the plant—leaves, bark, twigs, and stems—are rich in phenolic acids, flavonoids, hydrolyzable and condensed tannins, and volatile constituents. The chemical composition of *H. virginiana* extracts depends strongly on the extraction solvent and method, with water, aqueous ethanol, methanol, acetone, and ethyl acetate yielding various compound profiles. Among the most frequently reported compounds are gallic acid and its derivatives, catechin, epicatechin, hamamelitannin, quercetin and kaempferol glycosides, caffeoylquinic acids, and oligomeric and polymeric proanthocyanidins. Advanced analytical techniques—such as high-performance liquid chromatography coupled with electrospray ionization tandem mass spectrometry (HPLC-ESI-MS/MS), ultra-high-performance liquid chromatography coupled with mass spectrometry using quadrupole time-of-flight (UHPLC-MS-qToF), and gas chromatography coupled with mass spectrometry (GC-MS)—have enabled precise identification and quantification of these metabolites. Additionally, fractionation methods have facilitated the isolation of complex hydrolyzable tannins. The marked variation in chemical profiles based on extraction parameters highlights the need for standardized methodologies in phytochemical research. 

Leaf extracts, particularly those obtained using hot water or acetone–water mixtures, demonstrated the greatest diversity of phenolic compounds. Duckstein and Stintzing used HPLC-ESI-MS/MS analysis to identify several derivatives of caffeic and quinic acids—namely, 3-, 4-, and 5-caffeoylquinic acids—as well as flavonoid glycosides, including quercetin rutinoside, kaempferol hexoside, and kaempferol–galloyl hexoside [24]. Amêndola et al. demonstrated that methanolic leaf extracts also contained a wide array of bioactive compounds, including chlorogenic, ferulic, caffeic, ellagic, and salicylic acids, along with flavonoids such as apigenin, luteolin, and myricetin, and their respective glycosides (e.g., luteolin-7-o-glucoside, quercitrin, and rutin) [25]. Piazza et al., while studying glycolic extracts from twigs and bark, identified a broad spectrum of flavan-3-ols, including catechin, epicatechin, gallocatechin, and epigallocatechin, as well as the hydrolyzable tannin hamamelitannin. Using ultra-performance liquid chromatography coupled with triple quadrupole tandem mass spectrometry (UPLC/QqQ-MS/MS), they also detected additional phenolic acids such as ferulic, gallic, and protocatechuic acids, along with flavonoids like quercetin, isorhamnetin, and naringenin [26]. Wang et al. investigated water–ethanol extracts using HPLC-UV analysis, which confirmed the presence of gallic acid and hamamelitannin in both bark and leaves [27].

In turn, the fractionation of aqueous ethanol extracts has confirmed the presence of pentagalloyl glucose and tannic acid. UHPLC-MS qToF analysis further revealed a comprehensive profile of gallotannins in leaf extracts, spanning mono- to decagalloyl hexoses, including 1-o-galloyl-β-d-glucose and hamamelitannin [28]. Furthermore, Djapić demonstrated the presence of additional compounds in methanolic leaf extracts, including chlorophyll catabolites such as bilin-type and bilinone-type degradation products, as determined by HPLC analysis [29].

In addition to polyphenols, volatile compounds represent another class of phytochemicals that have been studied in *H. virginiana*. Engel et al. performed a steam distillation and water extraction of leaves and bark, which enabled the identification of over 170 volatile constituents. These included aliphatic hydrocarbons, alcohols, aldehydes, ketones, and esters. The chemical composition of the volatile fraction varied depending on the raw material used. In the leaf extracts, the major components are hydrocarbons (62.85%), monoterpenes (7.36%), terpenes (3.94%), and aldehydes (3.79%), followed by esters (0.95%), alcohols (0.86%), and ketones (0.83%). In comparison, bark extracts contain hydrocarbons (45.42%), terpenes (21.17%), monoterpenes (8.34%), alcohols (5.31%), aldehydes (6.14%), ketones (1.55%), esters (0.59%), and phenylpropanoids in significant quantities (over 7%) [30]. Cheesman et al., using GC-MS analysis, also detected a variety of alcohols and ethers containing aromatic rings and exhibiting potential biological activity [31]. 

**Figure 2:**
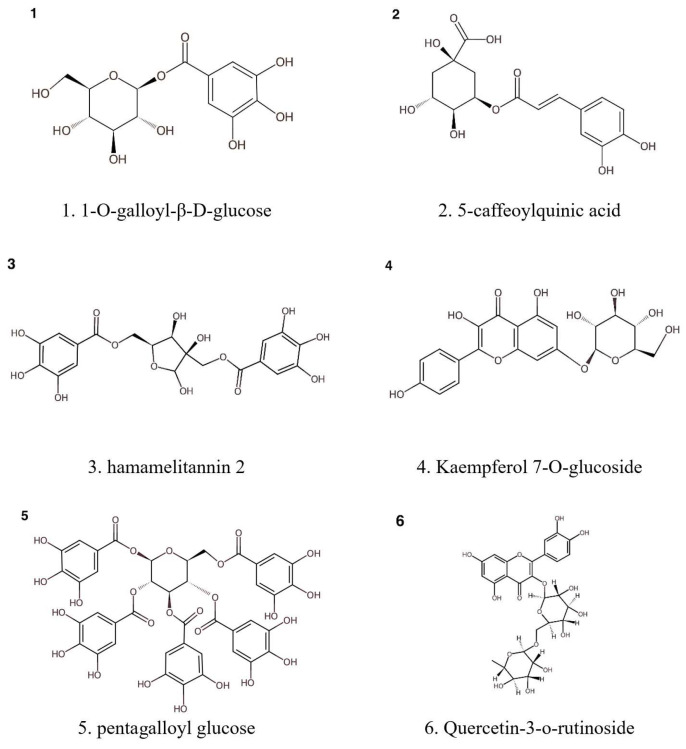
Example of some representatives polyphenolic compounds identified in *H. virginiana* extracts.

Figure 2 illustrates some representatives of specific classes of polyphenolic compounds identified in *H. virginiana* extracts. Table 1 summarizes the current findings on the phytochemical constituents of *H. virginiana*, with emphasis on solvent, extraction method, compound identification, and analytical techniques.

## 3. Biological Activity of *H. virginiana*

### 3.1. Traditional Uses and Regulatory Status of H. virginiana in Europe

The leaves and bark of *H. virginiana* have a long-standing history of use in traditional medicine and herbal remedies, particularly among Native American tribes. They utilized witch hazel preparations for a variety of skin-related ailments, including inflammation, wounds, insect bites, and irritation caused by poison ivy (*T. radicans*). In folk medicine, witch hazel extracts have been valued for their astringent, anti-inflammatory, and soothing properties. They have been traditionally employed to manage skin disorders such as eczema, psoriasis, and general skin dryness, as well as to alleviate minor injuries, razor burn, and local irritation. Furthermore, a decoction of the bark, due to its astringent properties, has traditionally been used to treat hemoptysis, hematemesis, and other types of hemorrhages, as well as diarrhea, dysentery, excessive mucous discharges, and hemorrhoids [2,5,40]. 

Beyond North America, the therapeutic use of *H. virginiana* has also become widespread in Europe. In countries such as Poland and Ukraine, witch hazel-based preparations have been used in ethnobotanical practices to treat inflammatory skin conditions and promote skin regeneration. Similarly, in the South Balkan and Eastern Mediterranean Region, traditional medicine has incorporated witch hazel for centuries, particularly in topical applications aimed at reducing swelling, redness, and irritation associated with minor dermatological complaints. Distilled witch hazel has traditionally been used to temporarily soothe eye irritation caused by dryness or environmental factors such as wind and sunlight [6,7,8].

The European Medicines Agency (EMA) has published two monographs on witch hazel—one for the bark and the other for the leaves. Both publications contain documented information on the medicinal use of the extracts. The monographs provide details on possible herbal preparations derived from raw materials, including the following: dried, comminuted herbal substance; tincture (fresh leaves) (ratio of herbal substance to extraction solvent 1:10, extraction solvent: ethanol 45% *v*/*v*); liquid extract (fresh leaves) (DER 1:1, extraction solvent: ethanol 45% *v*/*v*); liquid extract (DER 1:1, extraction solvent: ethanol 30% *m*/*m*); liquid extract (DER 1:2, extraction solvent: ethanol 60% *v*/*v*); and dry extract (DER 5–7.7:1, extraction solvent: ethanol 30% *m*/*m*). The documents also specify the forms and routes of safe administration (cutaneous, oromucosal, rectal, and anorectal) and indicate that the therapy is safe, with little or no side effects other than skin irritation, and no risk of overdose [41,42]. The reports also establish traditional therapeutic indications based on well-established usage for both pharmaceutical materials (cortex and folium): for the relief of minor skin inflammation and dryness, the symptomatic relief of itching and burning associated with hemorrhoids, and as a mouth and throat rinse to relieve minor inflammation of the oral mucosa. The authors specify that these products are traditional herbal medicinal products intended for use in the specified indications exclusively based on long-standing use [41,42].

The European Scientific Cooperative on Phytotherapy (ESCOP) has also issued its own specifications for these two pharmaceutical raw materials. The authors of the reports focused on similar aspects, such as preparation from plant material, forms of administration, applications, and safety, also adding information on the content of active compounds. They brought together relevant data on the possible physiological roles of *Hamamelis* leaf and bark extracts and their major constituents [43,44].

### 3.2. Literature-Based Insights into the Skin-Related Bioactivity of H. virginiana

In the context of potential benefits for skin health, several biological activities of *H. virginiana* extracts are particularly important to investigate, including antimicrobial, antioxidant, anti-inflammatory activities, wound healing effects, and UV protection. The antioxidant action helps neutralize free radicals, which are a major contributor to skin aging and cellular damage. Anti-inflammatory properties are also crucial, as they help prevent chronic inflammation that accelerates skin aging. Moreover, antimicrobial activity is especially relevant for products aimed at acne-prone or sensitive skin, where controlling microbial balance is essential. The extract’s potential to promote skin cell regeneration and support wound healing is equally important, as these processes are vital for maintaining healthy, resilient, and youthful-looking skin. Finally, UV-protective properties can help shield the skin from photoaging and other harmful effects of sun exposure.

**Figure 3:**
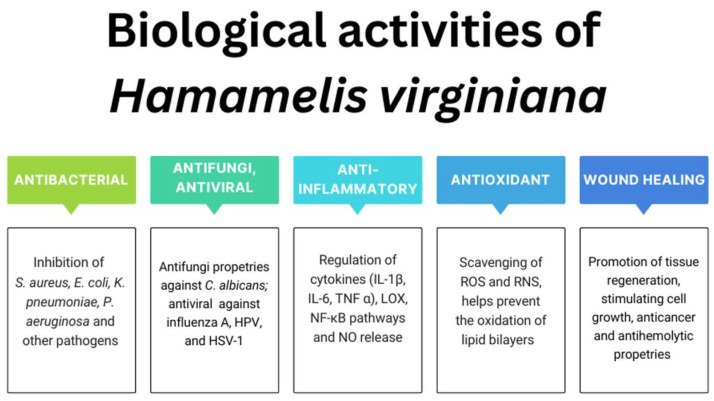
The main biological activities studied for *H. virginiana* extracts.

Figure 3 presents the main directions of action studied in the case of *H. virginiana* extracts.

#### 3.2.1. Antibacterial Effects

Due to its unique chemical composition, *H. virginiana* appears to be a promising antibacterial agent, and its antibacterial and antifungal properties have been extensively studied, covering both Gram-negative and Gram-positive bacteria. It should be noted that these two types of bacteria differ significantly in their cell wall structures, and therefore, distinct molecular mechanisms are involved in the bactericidal and bacteriostatic effects against Gram (−) and Gram (+) strains.

The most common parameter used to assess antibacterial activity is the minimum inhibitory concentration (MIC), which is the lowest concentration that shows no visible bacterial growth in broth. In agar-based assays, activity is often measured by the zone of inhibition (ZOI), defined as the radius of the clear area surrounding a disk infused with the antimicrobial agent. Additionally, the minimum bactericidal or fungicidal concentration (MBC/MFC), representing the lowest concentration that reduces microbial viability by 99.9%, is sometimes evaluated. 

Numerous studies have confirmed the effectiveness of both the leaves and bark of *H. virginiana* against various pathogens, including antibiotic-resistant strains. Cheesman et al. tested leaf extracts obtained using solvents of varying polarity against a sensitive bacterial strain and clinical isolates expressing extended-spectrum beta-lactamases (ESBLs), including *Escherichia coli*, ESBL *E. coli*, *Klebsiella pneumoniae*, ESBL *K. pneumoniae*, *Staphylococcus aureus*, and methicillin-resistant *S. aureus* (MRSA). The ethyl acetate, hexane, and chloroform extracts showed no activity. However, the water and methanolic extracts demonstrated antibacterial effects, with zones of inhibition (ZOI) ranging from 8 to 12 mm. The extracts were more effective against ESBL *E. coli* compared to sensitive *E. coli* and showed greater potency against MRSA than against *S. aureus*. Additionally, they evaluated the effects of combining these extracts with common antibiotics, finding additive interactions in three combinations: aqueous or methanolic extracts with tetracycline against MRSA, and methanol extract combined with chloramphenicol against *E. coli*. It is worth noting that additive interactions enhance efficacy, which may allow for lower antibiotic doses and thereby help minimize the risk of side effects [31]. The same experimental protocol was used to test the activity of the extracts against several *Staphylococcus* spp. (*S. oralis*, *S. pyogenes*, *S. mutans*) and *Streptococcus* spp. (*S. epidermidis*, *S. aureus*) strains. Similarly to previous findings, only the water and methanolic extracts showed activity. These extracts inhibited the growth of *S. oralis*, *S. pyogenes*, *S. epidermidis*, and *S. aureus* in both semi-solid and liquid cultures. However, *S. mutans* exhibited near-complete resistance to *H. virginiana*. Additive interactions were observed with the aqueous extract combined with chloramphenicol against *S. oralis* and with the methanol extract combined with ciprofloxacin against *S. aureus* [45]. The water and methanol extracts were also active against *P. aeruginosa*. Interestingly, combining these extracts with ciprofloxacin reduced the antimicrobial potency of each component when compared to their individual effects; therefore, these agents should not be used in combination [46]. 

The antimicrobial activity of the decoctions and methanol extracts from the leaves was also observed against anaerobic and facultative aerobic periodontopathic bacteria, including *Porphyromonas gingivalis*, *Porphyromonas asaccharolytica*, *Prevotella melaninogenica*, *Prevotella intermedia*, *Fusobacterium nucleatum*, *Capnocytophaga gingivalis*, *Veillonella parvula*, *Eikenella corrodens*, *Peptostreptococcus micros*, and *Actinomyces odontolyticus*. Similarly to the findings of Cheesman et al., Iauk et al. observed that methanol extracts demonstrated stronger antibacterial activity than decoctions, which are typically prepared by boiling in water [47]. The study also shows that rinsing with *Hamamelis* tea reduces initial bacterial colonization and increase the salivary pellicle thickness, thereby supporting oral hygiene and helping to prevent diseases such as caries and periodontitis [48]. In turn, Amêndola et al. investigated the antifungal effects of propylene glycol leaf extract, finding that it acts as a fungicide against *Candida* spp. (including *C. albicans*, *C. dubliniensis*, *C. glabrata*, *C. guilliermondii*, *C. krusei*, and *C. tropicalis*) [25,49]. However, its antibacterial activity against *Acinetobacter baumannii*, *E. coli*, *Enterococcus faecalis*, *K. pneumoniae*, *S. aureus*, and *S. mutans* was significantly lower, with even MIC values reaching over 50 mg/mL [25]. The study also examined the reduction in microbial biofilms after 5 min and 24 h of treatment using *H. virginiana* extracts. The extract was effective, but the results were strongly dependent on concentration and time. After 5 min, a concentration of 100 mg/mL led to an over 75% reduction in biofilm for most *Candida* spp., with the exception of *C. krusei*, which showed a 56% reduction. Less effectiveness was observed with bacterial strains, where biofilm reduction ranged from 52% to 79% at 100 mg/mL, except for *E. coli*, where 50 mg/mL resulted in a 75% reduction in biofilm. After 24 h, a concentration of 12.5 mg/mL reduced the biofilm formation of *Candida* spp., *E. coli*, and *K. pneumoniae* by over 79%. The least effective result was observed with *E. faecalis*, where 50 mg/mL produced a biofilm reduction of 91%. Both *S. aureus* and *S. mutans* showed a reduction of 93% at a concentration of 25 mg/mL [25]. The effectiveness of an alcoholic extract from *H. virginiana* in inhibiting the biofilm of clinical isolates of *S. aureus* was also demonstrated by Pereira et al. [50]. 

Rasooly et al. conducted research on the antibacterial properties of the commercial product whISOBAX (witch hazel bark ethanolic-water extract). They found that it suppresses bacterial growth and inhibits pathogenic mechanisms, including biofilm formation and toxin production. The greatest activity was observed against *S. epidermidis*, *S. aureus*, *Enterococcus faecium*, and *E. faecalis*, followed by *Acinetobacter baumannii* and *Klebsiella pneumoniae*. In contrast, the effects on *Escherichia coli*, *Pseudomonas aeruginosa*, *Streptococcus agalactiae*, and *Streptococcus pneumoniae* were significantly weaker. Furthermore, the extract acted synergistically with linezolid and chloramphenicol and showed cumulative effects with vancomycin and amikacin against *S. aureus* [51]. Additionally, its combination with green tea enhanced antibacterial activity against *S. epidermidis* and *S. aureus* [52]. Interestingly, despite its inhibitory effect on the growth and biofilm formation of pathogenic bacteria, whISOBAX has been found to stimulate the probiotic bacterium *Lactobacillus plantarum* in a nutritionally limited environment, while also sustaining its growth in a nutrient-rich environment [53]. It also protected against cell death induced by aerobic growing conditions [54]. Therefore, the authors concluded that it may support the maintenance of a healthy microbiome in the organism. 

The literature data on the antibacterial and antifungal effects of *H. virginiana* extracts are summarized in Table 2.

The antibacterial activity of witch hazel has been utilized in complex formulations. It was found that witch hazel bark extract (whISOBAX) enhances the antibacterial effects of commercially available teat dips, which can help prevent bacterial infections in dairy cattle [58]. Furthermore, a formulation with *Hamamelis* distillate and urea showed an inhibitory effect on *S. aureus* and *Candida albicans* [12]. In turn, hazel leaf extract in combination with *Krameria lappacea* root and *Salix alba* bark was used to develop a novel formulation with potential application in managing symptoms associated with seborrheic dermatitis. Both alone and when in the mixture, hazel extract was effective against MSSA, MRSA, *Bacillus cereus*, *Enterococcus faecalis*, and *Salmonella enterica*, and it showed no cytotoxicity on human keratinocytes [39]. Interestingly, Nardini et al. showed that extracts of *H. virginiana* may be useful in antimicrobial photodynamic therapy, as it exhibits photosensitizing properties combined with low toxicity to fibroblast cells and could therefore serve as an alternative to conventional photosensitizers [59].

In addition, attempts have also been made to develop antibacterial materials containing *H. virginiana* extract with potential applications such as wound dressings. However, these efforts have yet to yield successful results. For example, Solis-Arevalo et al. investigated the antibacterial and antifungal properties of leaf extract on its own and observed a significant inhibition of *Staphylococcus aureus*, *Candida albicans*, and *Pseudomonas aeruginosa*. Nevertheless, when they incorporated the extract into schizophyllan-based membranes, they did not observe any antibacterial effect [60]. Similarly, a lack of antibacterial activity was observed by Molin et al., who developed a bacterial cellulose membrane with aqueous and glycolic extracts of witch hazel and tested it against *S. aureus*, *E. coli*, and *C. albicans* [61]. The authors suggested that the concentration of active compounds in the materials prepared in this way is too low. On the other hand, newly developed bio-packaging material based on linseed mucilage with incorporated *H. virginiana* leaf extract effectively decreased foodborne pathogens, including *L. monocytogenes*, *S. Typhi*, *S. aureus*, and *E. coli*, with zones of inhibition ranging from 19.50 to 22.50 mm [55]. 

The antiviral efficacy of *H. virginiana* was also documented. Theisen et al. demonstrated the antiviral effects of bark and leaf extracts (60% ethanol), fractions containing tannins of different molecular weights, and individual tannins against influenza A virus (IAV) and human papillomavirus (HPV). They found that the antiviral effect of the leaf extract was similar to that of the bark extract, but the latter demonstrated lower cytotoxicity. High molecular weight condensed tannins, obtained via ultrafiltration, exhibited the highest activity. This fraction, along with the bark extract, inhibited the early stages and, to a lesser extent, later stages of the IAV life cycle and also prevented HPV attachment. Interestingly, high molecular weight tannins inhibited both IAV receptor binding and neuraminidase activity. In contrast, low molecular weight compounds, such as gallic acid, epigallocatechin gallate, and hamamelitannin, inhibited neuraminidase but not hemagglutination [32]. Hemagglutinin and neuraminidase are glycoproteins that play essential roles in the infection cycle, facilitating the initial attachment of the virus to the host cell and aiding in the release of newly formed viral particles from the host cell, respectively. Notably, the activity of the fractions and the extract was superior to that of the individual isolated compounds [32]. The fraction containing oligomeric and polymeric proanthocyanidins isolated from the hydroethanol extract of *H. virginiana* bark exhibited significant antiviral activity against herpes simplex virus type 1 (HSV-1) [62]. On the other hand, a standardized dry extract of *H. virginiana* leaf, obtained using 50% ethanol, showed no anti-HIV activity in infected human lymphocytic cells [63]. 

#### 3.2.2. Anti-Inflammatory Activity

Anti-inflammatory activity is another important feature of plant extracts in the context of skin health. In addition to directly soothing inflammatory conditions, anti-inflammatory agents can help counteract chronic low-grade inflammation, known as ‘inflammaging,’ by reducing the production of pro-inflammatory cytokines and oxidative stress. The effects of *H. virginiana* on various inflammatory mediators have been extensively studied, primarily using cell models with induced inflammation.

Piazza et al. investigated the potential of a standardized bark extract against eczema (AD) using human keratinocytes (HaCaT cells) induced with various cytokines including TNF-α (tumor necrosis factor alpha), IL-4 (interleukin 4), and a mixture of TNF-α/IL-4 and TNF-α/IFN-γ (interferon gamma) [64]. They found that the extract suppressed the release of mediators related to skin autoimmunity—IL-6 and IL-17C—and allergy—TSLP (thymic stromal lymphopoietin), IL-6, CCL26 (chemokine (C-C motif) Ligand 26), and MMP-9 (matrix metalloproteinase). All of these biomarkers are important contributors to the pathogenesis of the inflammatory processes in AD. However, the activity of the extract was only partially attributed to the hamamelitannin content, as this compound affected only TSLP and CCL26 and showed no inhibitory effect on the other factors investigated. The mechanism of action of the extract involved the disruption of NF-κB (nuclear factor kappa-light-chain-enhancer of activated B cells)-driven transcription. Additionally, it inhibited the proliferative effects of IL-4 and restored the expression of K10, a protein marker of skin differentiation [64]. In another study, Piazza et al. found that the extract decreased IL-6 and IL-8 levels in HaCaT cells infected with *Cutibacterium acnes* and IL-8 levels in HaCaT cells stimulated with TNF-α. Although it showed no inhibitory effect on bacterial growth, the observed anti-inflammatory properties could be beneficial in acne treatment. The results suggest that the biological activity of the extract is associated with its abundance of epicatechin-3-gallate [26].

In turn, Amêndola et al. evaluated the effect of the *H. virginiana* extract leaf on murine macrophages (RAW 264.7 cell line) stimulated with lipopolysaccharide (LPS) and found that the propylene glycol extract suppressed the production of IL-1β and TNF-α. The extract also reduced nitric oxide (NO) levels [25]. It is worth mentioning that although NO supports controlling infections by helping to eliminate pathogens through its antimicrobial and cytotoxic effects, excessive or prolonged NO production can contribute to tissue damage and promote chronic inflammation. 

The anti-inflammatory activity of *H. virginiana* can also be associated with the inhibition of lipoxygenase (LOX), an enzyme that plays a key role in the metabolism of arachidonic acid and contributes to the production of leukotrienes and prostaglandins. This was documented in a study by Hartisch et al., who observed that galloylated proanthocyanidins isolated from the extract suppressed 5-LOX activity [65]. In turn, Manville et al. stated that the dual anti-inflammatory and analgesic effects of witch hazel bark extract may result from the polymodal modulation of multiple potassium channel types [66]. An in vivo study by Erdelmeier et al. also confirmed the anti-inflammatory activity of *H. virginiana*. They observed that proanthocyanidins from the hydroethanol extract from bark exhibited strong anti-inflammatory effects in croton oil-induced dermal inflammation in mice [62]. In the study by Liu et al. [67], it was demonstrated that witch hazel inhibited the TNFα-induced expression of cytokines IL-1α, IL-1β, IL-8, and PGE2 (prostaglandin E₂) in HaCaT cells. The anti-inflammatory effect was further confirmed ex vivo in skin tissue with induced inflammation using a commercially available Cell Stimulation Cocktail, as the extract significantly decreased the levels of IL-6, IL-17A, TNFα, and IFN-γ compared to the control. Additionally, an analysis of skin barrier function markers, such as loricrin and transglutaminase-1, showed notable increases compared to the inflamed control, indicating improvements in skin integrity [67]. However, it should be noted that no data is given regarding the type of extract and even the part of plants taken for investigation. 

The data regarding the anti-inflammatory activity of *H. virginiana*, including experimental models and observed effects, are compiled in Table 3.

#### 3.2.3. Wound Healing Properties and the Other Effects on Skin

Wound healing is a complex, multi-stage process involving hemostasis, inflammation, cell proliferation, and tissue remodeling. After bleeding is stopped, an inflammatory response is triggered to prevent infection. In the next stage, there is increased cell proliferation, where new tissue and blood vessels form. Finally, in the remodeling phase, the tissue strengthens and matures [69]. 

Antibacterial activity is a critical feature in wound healing, as it helps create a microorganism-free environment and prevents wound infections. Similarly, anti-inflammatory action is essential to suppress prolonged inflammation, which is undesirable because it can lead to excessive tissue breakdown and inhibit effective tissue repair [70]. In this context, *H. virginiana*, which exhibits both types of activities, appears promising.

Another aspect to consider in wound healing is the regulation of proteolytic and oxidative enzymes at the wound site. Proteolytic enzymes, such as matrix metalloproteinases (MMPs), help clear damaged tissue and enable cell migration, while oxidative enzymes contribute to pathogen defense by producing reactive oxygen species (ROS). On the other hand, excessive enzyme activity can degrade the extracellular matrix (ECM) and, therefore, harm healthy tissue. Thus, maintaining an appropriate balance of these enzymes is crucial for optimal healing [69,71]. 

Several studies describe the effects of *H. virginiana* extracts on various aspects related to wound healing. Dezena and da Silva, who investigated the impact of a tincture prepared by percolation with 70% ethanol (10% *w*/*v*) on dewaxed and hydrated skin fragments from Wistar rats, found that it exhibited significant proteolytic activity on collagen and elastic fibers but did not affect epithelial and connective tissue cells, epidermal attachments, pigments, or granules [72]. Thring et al. observed that the water extract from the aerial parts and the distillate inhibited elastase, and additionally, the water extract showed anti-collagenase activity [68,73]. This type of activity could also be significant in anti-aging activity. Furthermore, polyphenols from *H. virginiana* stems, used in the preparation of collagen sponges for chronic wound dressings, demonstrated a suppression of two major enzymes that impair the wound healing process—myeloperoxidase (MPO) and collagenase [74]. Rocasalbas et al. used the phenolic fraction, consisting of a mixture of proanthocyanidin dimers and hydrolysable tannins (hamamelitannin, pentagalloyl glucose, and methyl gallate) isolated from stems, to develop bioactive hydrogel dressings. The hydrogels inhibited the growth of *S. aureus* and *P. aeruginosa*, bacteria which are commonly found in chronic wounds, and demonstrated inhibitory capacity against myeloperoxidase and collagenase [75]. It was also observed that the proanthocyanidins isolated from bark significantly increased the proliferation of human skin keratinocytes [34]. 

The protective effects of *H. virginiana* extract on skin have also been reported. Ramos et al. indicate that *H. virginiana* extract has potential as an antisolar agent, as evidenced by spectrophotometric measurements of sun protection factors and UV absorption [76]. In turn, Choi et al. found that *H. virginiana* can be a valuable ingredient in anti-pollution skincare products, as the extract from its stem and leaves lowered Ca²⁺ influx in HaCaT cells stimulated by particulate matter (PM2.5). The mechanism of action involved proteinase-activated receptor-2 (PAR-2), NF-κB, and occludin. The extract supported HaCaT cells in recovering from DPM-induced damage. The increased levels of PAR-2 (A) and NF-κB (B) induced by DPM were reduced to, or below, normal levels. Additionally, the decreased level of occludin caused by DPM returned to a normal state. This effect was attributed to hexagalloylglucose, isolated from the extract, which alone exhibited this type of activity [77].

In another study, the fractions from witch hazel bark rich in galloylated tannin showed anticancer potential against melanoma, with only mild cytotoxic effects on human skin fibroblasts and keratinocytes. Additionally, it was found to act as an antihemolytic agent, protecting red blood cells from hemolysis induced by oxidative stress [33].

#### 3.2.4. Antioxidant Activity

Antioxidant activity is the most commonly studied type of action in the case of plant extracts because its ability to scavenge free radicals can support the skin’s defense mechanisms against oxidative stress. This is particularly important in protecting the skin from premature aging, inflammation, and damage caused by environmental factors such as UV radiation and pollution. The antioxidant properties of *H. virginiana* were documented in many studies based on common chemical tests including DPPH, ABTS, and ORAC [25,26,57,78]. *H. virginiana* has also been found to actively scavenge singlet oxygen, superoxide anion radicals, hydroxyl radicals, and peroxynitrite, and it has been shown to prevent the oxidation of lipid bilayers [79,80,81]. Cells-based assay confirmed the antioxidant effects of both extracts and isolated components. It has been found that *H. virginiana* protects murine dermal fibroblasts against oxidative stress [79] and decreased reactive oxygen species (ROS) levels in H_2_O_2_-stimulated HaCaT [26]. However, this effect was not associated with an influence on catalase, one of the main enzymes responsible for maintaining redox balance in the cell, as no impact on the enzyme’s activity was observed [26,68]. The direct antioxidant effects of the water extract from the aerial parts was also demonstrated in Caco-2 and HepG2 cells, as shown by the Antioxidant Power 1 (AOP1) assay, which measures the ability to neutralize intracellular free radicals produced by photo-induction. This effect was associated with the modulation of the ARE/Nrf2 (antioxidant response element/nuclear factor erythroid 2–related factor 2) transcriptional pathway, which regulates the expression of numerous proteins involved in cellular antioxidant systems [82]. In turn, Liu et al. investigated the efficacy of witch hazel in mitigating UVA-induced oxidative stress and found a significant reduction in ROS levels when skin samples were pretreated with witch hazel formulations. Furthermore, treatment with the WH formula resulted in a significant decrease in 4-hydroxynonenal, a byproduct of the oxidation of sebum and keratinocyte cell membrane lipids, which had been significantly increased by UVA exposure [67].

#### 3.2.5. In Vivo Studies

Although in vivo studies on *Hamamelis* are limited, several investigations have examined its skin-related effects. Hughes-Formella et al. found that a topical lotion containing a 10% distillate of *H. virginiana* effectively reduced erythema induced by UV irradiation [83,84]. Furthermore, its extract has been effective in treating accidental skin injury caused by sodium hypochlorite solution during dental treatment. The extract alleviated burning and tenderness, and, after three months, reduced skin discoloration [85]. In turn, Wolff and Kieser assessed the clinical effects of a *Hamamelis* ointment containing a distillate of leaves and bark for treating inflammation and minor skin injuries in young patients up to the age of 11. They reported that ratings of the treatment by physicians and parents were similar to or even better than those for dexpanthenol [86]. A randomized controlled prospective cohort study conducted by Veronese et al. demonstrated that a cream for the eyelids and eye contour area with *H. virginiana* bark extract improved symptoms of eyelid dermatitis [87]. The anti-inflammatory effectiveness of oil-in-water emulsions containing *H. virginiana* distillate was also tested by Korting et al. in humans with inflammation induced by UV irradiation and cellophane tape stripping. They found that the emulsions reduced UV-induced erythema [88]. The emulsion also reduced itching and erythema in patients with atopic eczema; however, its effect was lower than that of hydrocortisone cream [89]. Moreover, a retrospective observational study by Trüeb, which included 1373 of patients, showed the effectiveness of witch hazel-based shampoo and tonic for the treatment of sensitive scalp irritation [17].

A preliminary in vivo evaluation of *Hamamelis* procyanidins further supports their skin benefits. A semi-solid formulation containing 1% *Hamamelis* procyanidins was tested for its ability to prevent irritant contact dermatitis induced by sodium lauryl sulfate (SLS) [34]. SLS irritation caused an increase in transepidermal water loss (TEWL), indicating local barrier disruption, but treatment with *Hamamelis* procyanidins significantly reduced this TEWL increase. Preventing TEWL is crucial for maintaining healthy skin because loss of moisture leads to dryness and irritation. An impaired skin barrier also makes the skin more susceptible to environmental aggressors, allergens, and pathogens, which can trigger inflammation and various skin disorders. Furthermore, pretreatment with the *Hamamelis* formulation clearly reduced clinical signs of inflammation. These findings were corroborated by colorimetry measurements assessing skin redness: SLS-induced irritation significantly increased erythema, which was noticeably reduced following treatment with procyanidins [34].

There are also a few studies concerning the effects of *H. virginiana* when taken orally. Natella et al. investigated the activity of aerial parts *H. virginiana* extract (Hamaforton™ capsule with 300 mg of the extract) on gene dysregulation induced by UVA radiation in human dermal fibroblasts, using a combination of in vivo and ex vivo experiments. In the first part of the study, 12 healthy volunteers received either Hamaforton™ or a placebo in a randomized, blinded crossover trial. In the next phase, serum containing Hamaforton™ metabolites—including 4-O-methyl gallic acid, 4-O-methyl gallic acid sulfate, and trimethyl gallic acid glucuronide—was used to enrich the culture medium of dermal fibroblasts exposed to UVA. Natella et al. observed an increase in the expression of ten genes associated in repair processes critical for the maintenance of skin integrity, suggesting that these metabolites may play a role in damage recovery [90]. In turn, Duwiejua et al. investigated the anti-inflammatory effects of a 70% ethanol leaf extract administered before and after the induction of rat paw edema. They found that *H. virginiana* was effective when administered after inflammation was induced, but it was only effective in the chronic state [91].

### 3.3. Biological Activity of Hamamelitannin

The most characteristic compound found in *H. virginiana* is hamamelitannin, which is present in the bark, stem, and leaves. This compound, together with other galloylated proanthocyanidins, contributes to the biological activity of the plants. Hamamelitannin isolated from *H. virginiana* exhibits potent free radical scavenging activity, as demonstrated in various chemical assays, including DPPH [92,93]. It has been identified as a strong inhibitor of 5-lipoxygenase (5-LOX), an enzyme involved in the progression of inflammation [65]. Masaki et al. found that hamamelitannin exerts protective effects against superoxide-induced damage in murine fibroblasts, showing greater activity than gallic acid, which constitutes its functional units [94]. Additionally, it demonstrated protective activity against UVB-induced cell damage in murine skin fibroblasts [95]. It has also been found to prevent the depolymerization of hyaluronic acid caused by oxidative stress [96].

The antimicrobial effects of hamamelitannin have also been reported. It has been found that hamamelitannin was effective against *S. aureus* isolates resistant to various antimicrobials and was able to inhibit biofilm production [97]. In a study by Cobrado et al. conducted in mice, it was demonstrated that subcutaneously implanted catheters previously soaked in a hamamelitannin solution exhibited significant infection control against *S. aureus*, *S. epidermidis*, and *Acinetobacter baumannii*. This compound reduced both the metabolic activity and biomass of the biofilm [98]. Hamamelitannin also showed antiviral efficacy against influenza A virus and human papillomavirus, with its mechanism of action involving neuraminidase inhibition [32]. The anti-inflammatory activity of hamamelitannin was studied by Piazza et al. in HaCaT cells stimulated with various inflammatory agents, including TNF-α, IL-4, TNF-α/IL-4, and TNF-α/IFN-γ. They observed a decrease in the expression of TSLP and CCL26, with no significant effect on IL-17C, MMP-9, or IL-6 [26,64].

## 4. A Global Regulation and Market Status of *H. virginiana*

*H. virginiana* has a long-standing tradition of use in both medicinal and cosmetic formulations across the globe. Its pharmacological properties, such as its anti-inflammatory, antioxidant, and wound-healing effects, have contributed to its popularity. However, the regulatory status and market presence of witch hazel in products vary significantly between regions such as the United States, the European Union, and parts of Asia.

In the USA, Hamamelis virginiana distillate (commonly referred as witch hazel water) is an over-the-counter (OTC) product regulated by the U.S. Food and Drug Administration (FDA). It is officially listed in the United States Pharmacopeia (USP) and is widely used in topical applications for minor skin irritations, insect bites, and hemorrhoidal relief. Cosmetic formulations containing witch hazel—toners, cleansers, and aftershaves—are classified as cosmetics under FDA regulation, provided that they do not exhibit any therapeutic effects, but act as an astringent ingredient [99,100]. 

In the European Union, witch hazel preparations can be used as a traditional herbal medicinal product (THMP) under Directive 2004/24/EC or as cosmetic products under Regulation (EC) No 1223/2009. Examples of THMPs include creams, rectal suppositories or liquid preparations for the treatment of minor inflammatory conditions of the skin. Regulatory assessment is carried out by national agencies or by the European Medicines Agency (EMA). In addition, the leaves and bark extracts of *H. virginiana* are registered for their cosmetic use in the European Cosmetics Ingredients database (CosIng) as astringents, skin and hair conditioners, and soothing ingredients [41,42,101]. 

In Asia, legal regulations are more dependent on the region and country. In countries like Japan, witch hazel extract is primarily used in the cosmetics industry, especially in facial care products. These products are regulated as quasi-drugs or functional cosmetics, depending on the national legislation of the Japanese Ministry of Health, Labor and Welfare (MHLW) [102]. For instance, witch hazel water is approved as a skin-conditioning agent. In China, products containing *H. virginiana* may fall under the regulatory oversight of the National Medical Products Administration (NMPA) [103], with the most recent focus predominantly being on the botanical ingredients of cosmetics, supporting the consumers demand for “clean beauty” solutions in these products. However, the formal recognition of using witch hazel in these regions as a traditional medicine is limited.

## 5. Conclusions

Our study showed that *Hamamelis virginiana* (witch hazel) demonstrates a wide range of biological activities that support its use in dermatology, particularly regarding skin health. Extracts from its bark and leaves show antibacterial, antifungal, antiviral, anti-inflammatory, antioxidant, and wound healing properties. It is effective against various bacteria, including antibiotic-resistant strains, and fungi like Candida, with synergistic effects alongside antibiotics. Clinical studies support its use in eczema, acne, UV-induced erythema, and sensitive scalp conditions. *Hamamelis* also promotes wound healing by modulating enzymes, reducing oxidative damage, and stimulating skin cell growth. Its antioxidant properties protect skin from oxidative stress and pollution. Taken together, current scientific evidence confirms the relevance of *H. virginiana* as a traditional herbal remedy. Its multifunctional bioactivity, wide availability, and favorable safety profile position it as a promising candidate for integration into modern dermatological and cosmetic formulations. The future potential of witch hazel lies not only in its traditional uses but also in novel applications supported by rigorous scientific validation. Therefore, despite these promising findings, several areas require further investigation.

The molecular mechanisms responsible for the biological effects of *H. virginiana* are worth investigating, with particular emphasis on the regulation of intracellular signaling pathways. Clarifying which signaling cascades are selectively activated or suppressed by its bioactive compounds could contribute to the identification of specific molecular targets. This knowledge would not only help explain its pharmacological actions—such as its anti-inflammatory, antioxidant, or antimicrobial effects—but could also aid in optimizing therapeutic applications and minimizing potential side effects. Variability in extract composition highlights the necessity for standardized extraction protocols and comprehensive phytochemical profiling. It is also worth including the flowers of this interesting plant in future studies, as they may prove to be a valuable source of beneficial phytochemicals. Additionally, while environmental and geographical factors are known to influence the phytochemical content of many medicinal plants, there are currently no published studies examining whether the efficacy or composition of *H. virginiana* extracts vary depending on the cultivation region. Addressing this gap could provide valuable insights for optimizing cultivation practices and ensuring consistent therapeutic quality. Furthermore, although preliminary clinical results are encouraging, larger and well-controlled trials are essential to confirm efficacy in specific skin conditions. Moreover, long-term safety and toxicity profiles of different formulations, especially at higher concentrations, remain insufficiently explored. Research into optimal delivery systems, such as emulsions and hydrogels, to improve the bioavailability of active compounds in topical applications is also warranted. Lastly, further studies on synergistic or antagonistic interactions with conventional antibiotics and skincare agents are critical to inform effective combination therapies. 

In conclusion, *H. virginiana* represents a multifaceted natural agent with significant potential for skin health and is deserving of continued translational and clinical research to fully harness its therapeutic benefits.

## Figures and Tables

**Table 1:** Chemical composition of *Hamamelis virginiana* L.

Extraction Solvent, Method	Plant Part	Compounds Identified	Identification	Ref
Water, maceration at 5 °C	Leaves	3-, 4- and 5 -caffeoylquinic acid, 3- and 5- *p*-coumaroylquinic acid, catechin, procyanidin trimer and dimer, gallic acid–quinic acid ester, caffeoylshikimic acid, quercetin (Q) and Q-galloyl hexoside, ellagic acid, Q- rutinoside, kaempferol (K) and K- hexoside, K- hexoside–deoxyhexoside, K-galloyl hexoside, mono-, hexa-, hepta- octa-, nona- and deca-galloyl hexose,	HPLC-ESI-MS/MS	[24]
Acetone–water (8:2), maceration at 5 °C	Leaves	3-, 4- and 5 -caffeoylquinic acid, quercetin–galloyl hexoside, quercetin rutinoside, kaempferol (K) and K- hexoside, K- hexoside–deoxyhexoside, K-galloyl hexoside, quercetin, hexa-, hepta-, octa-, nona- and decagalloyl hexose	HPLC-ESI-MS/MS	[24]
Glycol, no data on extraction method	Twigs and bark	4-hydroxybenzoic acid, vanillin, vanillic acid, protocatechuic acid, methyl gallate, ferulic acid, ellagic acid, isorhamnetin-3-*o*-glucoside, quercetin-3-*o*-glucoside, naringenin, catechin, epicatechin, gallocatechin, epigallocatechin, hamamelitannin	UPLC/QqQ-MS/MS	[26]
Water, acetonitrile ethanol, acetone (15%), sonication	Bark, twigs, leaves	hamamelitannin, catechin, gallocatechin, gallic acid	HPLC-UV	[27]
60% ethanol in water, maceration and ultrafiltration	Bark, leaves	hamamelitannin, pentagalloylglucose, gallic acid, tannic acid	LC (isolation)	[32]
Purified hot water, heating at 90 °C	Leaves	1-o-galloyl-β-d-glucose, gallic acid, galloyl-hexose a, c, d, hamamelitannin, tetra-, penta-, hexa- hepta-, octa-, nona-, and deca-o-galloyl-hexose	UHPLC-MS qToF	[28]
Maceration with acetone–water (7:3), fractionation from water	Stems	flavanol (catechin) monomers, proanthocyanidins, hydrolyzable tannins: hamamelitannin, methyl gallate, pentagalloyl glucose	LC-MS-MS	[33]
propylene glycol, maceration	Leaves	derivatives of gallic acid	HPLC-DAD	[25]
Isolation from polyvinylpyrrolidone/water	Bark	polysaccharides and procyanidins	GPC, GC -MS	[34]
Extraction with acetone–water (7:3); isolation from water	Bark	catechin, tannins (hamamelitannin), proanthocyanidins	1H-NMR, HPLC	[35]
Extraction with acetone–water (7:3); isolation from water	Bark	polymeric proanthocyanidins, epicatechin, epigallocatechin	GPC, HPLC, TLC	[36]
45 and 70% ethanol, isolation using ethyl acetate followed by LC	Leaves and bark	kaempferol, quercetin, trifolin, kaempferol-3-o-d-glucuronide, hyperin, quercituron/mikwelianin, caffeic, chlorogenic, gallic acids, hamamelitannin, cyanidine, delfinidine	Identification by melting point and UV-Vis spectra	[37]
Water distillation (volatile fraction)	Leaves and bark	about 175 (leaves) and 168 (bark) volatile compounds: homologous series of alkanes, alkenes, aliphatic alcohols, aldehydes, ketones, fatty acid esters	GC-MS	[30]
Water, maceration	Whole plant	gallic acid, hamamelitannin	HPLC-DAD, FL	[38]
50% ethanol in water, maceration under periodical stirring	Leaves	caffeic acid, carnosic acid, chlorogenic acid, ferulic acid, gallic acid, ellagic acid, salicylic acid, trans-p-coumaric acid, apigenin, catechin, chrysin, hyperoside, kaempferol, luteolin, luteolin-7-*o*-glucoside, myricetin, naringenin, pyrocatechol, quercetin, quercitrin, rutoside, vitexin	LC-MS	[39]
Water, methanol, ethyl acetate, hexane, chloroform, maceration under periodical stirring	Leaves	isobutyl ether, 3,5,5-trimethylhexanol, 2-ethyl-1-hexanol, 1-nonanol, menthol, 3-methoxy-3-methylbutanol, phthalane, nonanal, 2-ethyl-1-hexyl acetate, 2-heptyl-1,3-dioxolane, 5,6,7,8,9-octahydro-2h-benzo[a]cyclohepten-2-one, decanal, epoxy-cumene, 1,3-di-tert-butylbenzene, trans-2-decenal, camphene, 1,3-dioxolane-2-methanol, 2,2,4-trimethyl-1,3-pentanediol diisobutyrate, (e)-2-dodecen-1-al, 1,3-pentanediol, 2,2,4-trimethyl-, 1-isobutyrate	GC-MS	[31]

HPLC—high performance liquid chromatography; GC—gas chromatography; LC—liquid chromatography; DAD—UV-Vis detector; FL—fluorescence detector; MS—mass spectrometry; HPLC-ESI-MS/MS—high-performance liquid chromatography coupled with electrospray ionization tandem MS; UHPLC-MS-qToF—ultra-high-performance liquid chromatography coupled with MS using quadrupole time-of-flight; UPLC/QqQ-MS/MS—ultra-performance liquid chromatography coupled with triple quadrupole tandem MS; TLC—thin-layer chromatography; NMR—nuclear magnetic resonance; GPC—gel permeation chromatography.

**Table 2:** Antibacterial and antifungal activity of extracts from *H. virginiana*.

Material/Extract/Method	Antibacterial/Antifungal Effect	Ref
Leaf/water (w), methanol (m) 24 h of maceration with continuous mixing	MIC (µg/mL) *Escherichia coli* 3448 (w); 1173 (m) *ESBL E. coli* 1724 (w); 670 (m) *Staphylococcus aureus* 493 (w); 251 (m) *MRSA* 431 (w); 168 (m) *Klebsiella pneumoniae* 1724 (w); 1341 (m) *ESBL K. pneumoniae* 2463 (w); 1257 (m)	[31]
	MIC (µg/mL) *Staphylococcus oralis* 1478 (w); 838 (m) *Staphylococcus pyogenes* 1724 (w); 503 (m) *Staphylococcus mutants* 4925 (w) >10,000 (m) *Streptococcus epidermidis* 308 (w); 210 (m) *Streptococcus aureus* 493 (w); 251 (m)	[45]
	*Pseudomonas aeruginosa* 1724 (w); 587 (m) µg/mL	[46]
Leaf/propylene glycol (no detail on method)	MIC/MFC (mg/mL) *Candida albicans* 1.56/6.25, *Candida dubliniensis* 0.78/3.12, *Candida glabrata* 1.56/6.25, *Candida guilliermondii* 0.39/3.12, *Candida krusei* 0.19/3.12 *Candida tropicalis* 0.39/3.12.	[25]
	MIC/MBC (mg/mL) *Acinetobacter baumannii* 3.13/12.5, *Escherichia coli* 12.5/25, *Klebsiella pneumoniae* 12.5/12.5, *Enterococcus faecalis* >50 *Streptococcus aureus*, *S. mutans* >50	[25]
Leaves/water 10% decoctions	MIC (mg/L) *Porphyromonas asaccharolityca* 256 *P. gingivalis* (5) * 256, 1024, 1024, 8192, 16,384 *Prevotella melaninogenica* (2) * 256, 256 *Prevotella intermedia* 2048 *Fusobacterium nucleatum* ≥16,384 *Capnocytophaga gingivalis* 16,384 *Veilonella parvula* 4096 *Eikenella corrodens* 128 *Peptostreptococcus micros* (2) * 4096, 8192 *Actinomyces odontolitycus* (3) * 128, 256, 128	[47]
Leaves/methanol (Soxhlet)	MIC (mg/L) *Porphyromonas asaccharolityca* 128 *P. gingivalis* (5) * 64, 256, 256, 512, 2048 *Prevotella melaninogenica* (2) * 64, 64 *Prevotella intermedia* 512 *Fusobacterium nucleatum* 16,384 *Capnocytophaga gingivalis* 4096 *Veilonella parvula* 2048 *Eikenella corrodens* 32 *Peptostreptococcus micros* (2) * 2048, 2048 *Actinomyces odontolitycus* (3) * 32, 128, 32	[47]
Leaves/50% ethanol (1:6) maceration for 10 days, decantation and filtration	ZOI/MIC/MBC (mm/mg GAE/µL) MSSA 19.17/0.2494/0.4988 MRSA 16.83/0.2494/0.4988 *Bacillus cereus* 18.17/0.2494/0.4988 *Enterococcus faecalis* 17.67/0.4988/0.4988 *Salmonella enterica* 10.83/0.4988/0.4988 *Escherichia coli* 8.5/0.4988/0.4988 *Pseudomonas aeruginosa* 0/-	[39]
Commercial ethanolic fluid extract (leaves; 152 mg mL^−1^)	MIC (mg/mL) *Listeria monocytogenes* 1.18 *Staphylococcus aureus* and *S. typhi* 2.37 *Escherichia coli* 2.37	[55]
whISOBAX (commercial ethanolic bark extract)	MIC (µg/mL) *Staphylococcus epidermidis* 26 *Staphylococcus aureus* 26–104 *Enterococcus faecalis* 39–52 *Enterococcus faecium* 19–52 *Staphylococcus agalactiae* 1250–6667 *Staphylococcus pneumoniae* 2500–6667 *Acinetobacter baumannii* 156–208 *Klebsiella pneumoniae* 312–833 *Pseudomonas aeruginosa* 1667–5000 *Escherichia coli* 1250–10,000	[52]
Leaves (l) and bark (b)/boiling water for 5 min evaporation to dryness	ZOI/MIC (mg/mL) *Staphylococcus aureus* 15–16.5/0.4–0.8 (l); 13.7–16/10 (b) *Enterococcus faecalis* 12.2/0.3 (l); 13.8/10 (b) *Bacillus subtilis* 12.5/1.1 (l); 12.7/10 (b) *Escherichia coli* 16.5/0.4 (l); 11.0/10 (b)	[56]
No data/maceration with 50% ethanol and 6% glycerin	*Staphylococcus epidermis* ZOI 18 mm *Propionibacterium acnes* subsp. *acnes* ZOI 18 mm *Propionibacterium granulosum* ZOI 17 mm	[57]

ESBLs—clinical isolates expressing extended-spectrum-lactamases; MRSA—methicillin-resistant *Staphylococcus aureus*; MSSA—methicillin-susceptible *S. aureus*; *—number of strains; MIC—minimum inhibitory concentration; ZOI—zone of inhibition; MBC—minimum bactericidal concentration; MFC—minimum fungicidal concentration; l—leaves; b—bark; w—water extract; m—methanol extract.

**Table 3:** Anti-inflammatory activity of *H. virginiana* extracts.

Extract	Model	Observed Effect	Ref
Propylene glycol leaf 25, 50 and 100 mg/mL	Murine macrophages stimulated with LPS	↓IL-1β ↓ TNF-α	[25]
Propylene glycol leaf 25, 50 and 100 mg/mL	Murine macrophages	NO ↑	[25]
Bark glycolic extract 0.5–50 µg/mL	HaCaTs induced with TNF-α	↓IL- 17C (IC50 1.53 µg/mL) ↓ MMP-9 (IC50 1.11 µg/mL) ↓ IL-8 (IC50 38.93 µg/mL)	[26,64]
Bark glycolic extract 0.5–50 µg/mL	HaCaTs induced with TNF-α/INF -γ	↓ TSLP (4.33 µg/mL) ↓ IL-6 (IC50 2.70 µg/mL)	[64]
Bark glycolic extract 0.5-50 µg/mL	HaCaTs induced with TNF-α/IL-4	↓ IL-6 (IC50 2.70 µg/mL) ↓ IL-6 (IC50 = 21.30 µg/mL)	[64]
Bark glycolic extract 0.5-50 µg/mL	HaCaTs induced with IL-4	↓CCL26 (IC50 = 21.36 µg/mL)	[64]
Bark glycolic extract 5-250 µg/mL	HaCaTs infected with *C. acnes*	↓ IL-6 (IC50: 136.90 µg/mL ↓ IL-8 No impact on NO	[26]
Dried herb/water distillate Evaporated (25, 50, 100, µg)	Fibroblast induced with H_2_O_2_	↓ IL-8	[68]
Proanthocyanidins from bark/60% ethanol	Mouse ear treated with croton oil	↓ Inflammation	[62]

LPS—lipopolysaccharide; HaCaTs—human keratinocytes; MMP—matrix metalloproteinase; IL—interleukine; TNF-α—tumor necrosis factor alpha; IFN-γ—interferon gamma; NO—nitric oxide; TSLP—thymic stromal lymphopoietin; CCL26—chemokine (C-C motif) ligand 26.

## Data Availability

Data is contained within the article.
